# An Extensive Study of the Influence of Key Flow Variables on Printed Line Quality Outcomes during Aerosol Jet Printing Using Coupled Three-Dimensional Numerical Models

**DOI:** 10.3390/ma17133179

**Published:** 2024-06-28

**Authors:** Haining Zhang, Haifeng Xu, Lin Cui, Zhenggao Pan, Pil-Ho Lee, Min-Kyo Jung, Joon-Phil Choi

**Affiliations:** 1 School of Information Engineering, Suzhou University, Suzhou 234000, China; m160034@e.ntu.edu.sg (H.Z.); hf.xu.su@gmail.com (H.X.); cl@ahszu.edu.cn (L.C.); szxypzg@ahszu.edu.cn (Z.P.); 2 School of Mechanical and Aerospace Engineering, Nanyang Technological University, Singapore 639798, Singapore; 3 Department of 3D Printing, Korea Institute of Machinery & Materials, Daejeon 34103, Republic of Korea; pilho_lee@kimm.re.kr (P.-H.L.); mkjung@kimm.re.kr (M.-K.J.)

**Keywords:** 3D numerical model, discrete phase model, aerosol jet printing, three-dimensional, printed line electrical performance, printed line quality

## Abstract

A three-dimensional (3D) numerical model was developed to explore the intricate aerodynamic mechanisms associated with aerosol jet printing (AJP). The proposed approach integrates computational fluid dynamics and discrete phase modeling, offering a comprehensive understanding of the deposition mechanisms of the AJP process. Initially, numerical solutions of the governing equations were obtained under the assumptions of compressible and laminar flows, facilitating an analysis of certain key flow variables, in this case, the sheath gas flow rate and carrier gas flow rate across the fluid domain. Subsequently, incorporating a Lagrangian discrete phase model allowed a detailed examination of the droplet behavior after nozzle ejection, considering the influence of the Saffman lift force. Finally, experiments were performed to elucidate the influence of key flow variables on the printed width. Generally, the measured printed line morphology and corresponding line electrical performance exhibited close conformity with the numerical model, demonstrating that the proposed numerical model is important for making well-informed decisions during process optimization.

## 1. Introduction

Recently, aerosol jet printing (AJP) technology has risen to prominence as an innovative non-contact direct writing method in advanced manufacturing [1,2,3]. These methods are increasingly preferred over traditional syringe and ink-jet approaches due to their notable benefits [4,5]. AJP, in particular, stands out as it can be with a diverse array of functional inks, expanding its usability and significantly reducing the number of production steps required, thereby reducing chemical waste and lowering production costs [6,7]. As a result, AJP is acknowledged as an environmentally friendly and cost-effective manufacturing solution responsible for substantial progress in the field of printed electronics [8,9]. The adaptability and high resolutions offered by the AJP process are particularly appealing to the aerospace and electronics industries, which require the precise placement of advanced electronic components on compact surfaces with tight spacing patterns. For instance, Liu et al. and Zhao et al. utilized AJP technology to fabricate flexible sensors for diverse applications [10,11], Clifford et al. and Paulsen et al. produced integrated electronic circuits on 3D Structures or thin film with AJP technology [12,13,14], Serpelloni et al. and Cao et al. developed flexible and stable thin-film transistors by means of AJP technology [15,16]. Additionally, AJP shows promise as an AI-enhanced manufacturing method, offering precision, flexibility, and the capability for real-time monitoring, optimization, and quality control through the integration of artificial intelligence [2,17].

While AJP demonstrates distinctive advantages and diverse applications, challenges such as material instability and limited control over the printing process can significantly affect the line morphology, presenting major barriers to enhancing AJP technology. For example, Seifert et al. and Mahajan et al. experimentally demonstrated the significant influence of process parameters on aerosol jet printed line morphology [18,19]. Consequently, considerable research efforts have been devoted to refining the AJP technique. For instance, since the electrical functionality of microelectronics produced through AJP is greatly dependent on process parameters such as the sheath gas flow rate (SHGFR), carrier gas flow rate (CGFR), and standoff distance, numerous studies aimed to optimize these key influencing factors for particular uses [20]. Salary et al. found that line width increases proportionally with CGFR until it stabilizes [21,22], whereas Goth et al. reported contradictory findings, which they attributed to an overly high SHGFR causing inconsistent flow collimation during printing [23]. Likewise, while the SHGFR enhances the focusing ability of AJP, maximum focus is achieved only after the SHGFR surpasses a specific level [8]. The effect of adjusting the working height from 5 mm to 12 mm was also examined, revealing that an optimal distance is essential for achieving high-quality prints [19,24]. These studies suggest that sub-optimal settings of AJP parameters can lead to poor line quality, underlining the need for a thorough analysis of fluid dynamics within the printhead to improve control over the transport and deposition of the aerosolized ink.

In these cases, various theoretical methods were formulated to explain the aerodynamic behaviors during printing. Applying an analytical model, Chen et al. analyzed the velocity profile inside the microcapillary, highlighting its significant role along with the droplet size in influencing the forces acting on the particles [25].

Additionally, the relationship between droplet size and fluid shear rate greatly affects droplet alignment, prompting 2D computational fluid dynamics (CFD) modeling to study the impact of influencing factors on printing quality [26,27,28]. These studies identified the Saffman lift force as a crucial parameter in determining whether narrower line widths were achieved. With the focus of CFD-based research being the aerodynamic interactions within the printing channel, further comprehensive studies are required to examine how various influencing factors affect the overall printing quality. Consequently, extensive research based on the CFD model and discrete phase models (DPM) has been undertaken to examine the effects of the CGFR and SHGFR on line morphology [29,30]. Notably, an increase in the SHGFR enhances particle collimation at the nozzle tip, leading to finer lines. Conversely, a slight increase in the CGFR produced wider line widths [21,22]. Overall, the flow dynamics in the printhead provide insights into the mechanisms of aerosol transportation in AJP. However, with particle trajectories from the nozzle directly impacting certain line features, further investigations of the forces acting on the particles and their behaviors under varying conditions are crucial. This could aid in clarifying the particle deposition process and enhancing the control of AJP. Furthermore, the issue of droplet breakup during transportation, influenced by high relative velocities, remains unexplored, and explanations regarding particle trajectories upon nozzle exit are insufficient. Hence, there is a clear demand for a comprehensive 3D numerical model capable of systematically analyzing the effects of different printing parameters on the line morphology in AJP.

In this study, a three-dimensional (3D) numerical model integrating CFD and DPM was developed to explore the intricate aerodynamic mechanisms within AJP. Initially, numerical solutions were obtained for the governing equations under the assumptions of compressible and laminar flows, facilitating an analysis of key flow variables, in this case, the SHGFR and CGFR across the fluid domain. Then, a Lagrangian discrete phase model with a Taylor analogy breakup (TAB) model is incorporated to allow a detailed examination of droplet behavior after nozzle ejection, considering the influence of the Saffman lift force. Finally, experiments were performed to elucidate the influence of the analyzed key flow variables on the printed width. Different from previous research [21,22], this study investigates the forces acting on particles and their behaviors under varying conditions, especially particle trajectories after exiting the nozzle. Moreover, the issue of droplet breakup during transportation is also considered and explored in the developed model. The experiments further investigate the influence of CGFR and SHGFR on the electrical performance of the printed line. Generally, the measured printed line morphology and corresponding electrical performance exhibited close conformity with the numerical model, affirming the utility of the proposed model for informed parametric optimization decisions.

## 2. Experiments and Analysis

### 2.1. Experimental Methods

**Figure 1:**
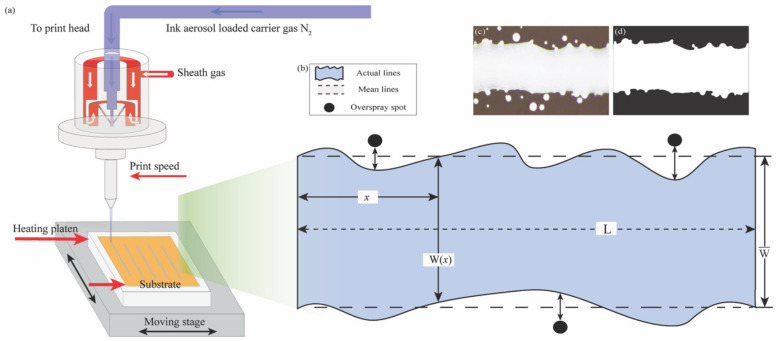
Illustration of the AJP technology: (**a**) Basic working principles of the AJP process, (**b**) mean line width definition, (**c**) captured original line sample image, and (**d**) captured line sample image with denoising.

Figure 1a outlines the fundamental operational methods of AJP technology employed in this study. To start with, an ultrasonic transducer facilitates the generation of ink droplets ranging in diameter from 0.5 µm to 6 µm from the ink bottle [21,25]. These atomized aerosols are subsequently conveyed to the printhead by a carrier gas flow. Then, within the print channel, a sheath gas flow envelops the aerosol, aiding in its acceleration. Upon reaching the moving platform, the ink stream accumulates, depositing a conductive line onto the substrate. Generally, the deposition process shows the direct effects of the particle forces and ink aerosol collimation on the printing resolution. Therefore, it is imperative to conduct a comprehensive investigation into the impact of the key gas flow parameters (SHGFR and CGFR) on the particle forces and the corresponding trajectories.

In this research, a nanoparticle silver ink was blended with deionized water at a 1:1 ratio and maintained at 20 °C for printing (Clariant^®^, Louisville, CO, USA). Pure nitrogen gas (99.99%) served as the sheath gas and carrier gas at 25 °C. The current for aerosol atomization was adjusted to 0.45 A, and the primary gas flow rates were determined in standard cubic centimeters per minute (sccm). Prior to printing, the polyimide substrates used in this study underwent three minutes of bath cleaning followed by one minute of a corona plasma ultrasonic treatment to enhance the substrate wetting behavior. Then, using a nozzle with a diameter of 150 µm, a single pass of a line sample was produced at a print speed of 1 mm/s; each designed point was subjected to five experiments for validation purposes. The detailed experimental setup of the AJP process is shown in Table 1.

### 2.2. Morphological Analysis

After experimentation, the characteristics of the produced lines were promptly assessed using an optical microscope, and Python code was developed for image processing. Figure 1b presents the geometric attributes of the produced line sample. In particular, the mean line width ($\bar{w}$) was determined as the average distance between the line edges. As depicted in Figure 1c,d, the overspray spots in the initial figure of a deposited line sample underwent pre-processing and transformation into a discretized binary form. Subsequently, the mean line width could be evaluated by identifying the actual line edge from the grayscale image. In this research, the average line width ($\bar{w}$) each experimental point was determined by measuring and averaging the widths of three printed line samples:

(1) $$\bar{w}=\frac{1}{N}\sum_{i=1}^{N}w_{i}$$

where $w_{i}$ represents the line width of the i-th column after discretization, and N represents the total number of columns along the discretized edge.

## 3. D-CFD Modeling of the Aerosol Flow

### 3.1. Modeling and Mesh Generation for 3D Geometry

**Figure 2:**
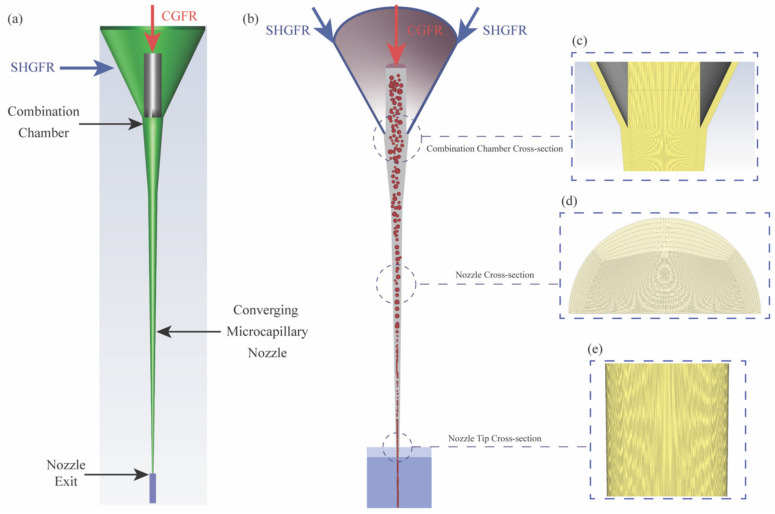
Modeling and mesh generation for the AJP printhead: (**a**) simplified 3D model of AJP, (**b**) 3D geometry meshing of the simplified AJP printhead, and mesh demonstration of (**c**–**e**) combination chamber, flow transport channel, and nozzle exit, respectively.

This study utilized a combination of tools, including a caliper, a digital microscope, and X-ray computed tomography, to measure the external and internal dimensions of the original 3D geometry of the printhead. Notably, the print channel converges from a wide inlet (diameter: 1.7 mm) to a narrower exit (diameter: 150 µcm) over a short length (4.2 cm), which significantly impacts the aerosol transportation process within the printhead. Following the determination of the original model, a simplified 3D model geometry was devised, as presented in Figure 2a. Then, as shown in Figure 2b, the simplified 3D CFD model underwent partitioning and subsequent discretization, resulting in 5,031,494 nodes dispersed across the 3D geometry. A refined mesh was specifically created to capture the particle-fluid interaction within a designated zone. This mesh was loaded into FLUENT (Ansys^®^, Canonsburg, PA, USA) for a detailed simulation. Figure 2c–e depicts the mesh distribution for three specially selected regions in the printhead, respectively.

### 3.2. Governing Equations

#### 3.2.1. Basic Theories of Governing Equations

The fundamental operational methods of ultrasonic AJP reveal its intricate nature as a multiphase flow problem. In AJP, the aerosol flow comprises three primary constituents: (1) solid nanoparticles suspended within a liquid medium, (2) forming ink droplets carried by a stream of carrier gas, which is (3) encased within a focused jet from a sheath gas stream and deposited onto a substrate. To assess the pressure distribution, fluid velocity, and density as well as the behavior of the aerosol particles upon exiting the nozzle, the density-based Navier–Stokes algorithm is employed in this study. This algorithm addresses the challenges of the high-speed compressible and Newtonian fluid flows involved here within the intricate geometry of the nozzle. For each simulation under steady-state conditions, converged solutions of the density, velocity, and pressure fields are obtained using the equations below for the continuity, momentum, energy, and state, respectively:

Continuity equation

(2) $$\frac{\partial \rho }{\partial t}+\nabla \cdot \left(\rho \overset{⃑}{\upsilon }\right)=0$$

Momentum equation

(3) $$\frac{\partial \left(\rho \overset{⃑}{\nu }\right)}{\partial t}+\nabla \cdot \left(\rho \overset{⃑}{\upsilon }\overset{⃑}{\upsilon }\right)=\nabla p+\nabla \cdot \left(\mu \nabla \overset{⃑}{\nu }\right)+S_{M}$$

Energy equation

(4) $$\frac{\partial \left(\rho e\right)}{\partial t}+\nabla \cdot \left(\rho e\overset{⃑}{\nu }\right)=-p\nabla \cdot \overset{⃑}{v}+\nabla \cdot \left(k\nabla T\right)+\Phi +S_{E}$$

State equation

(5) $$p=\rho RT\;\;and\;\;e=C_{v}T$$

where *ρ* (kg/m^3^), *T* (K), *P* (Pa), *e* (J/kg), *μ* (Pa·s) *K* (W/(m·k)), and $\vec{v}$ (m/s) represent the fluid density, fluid temperature, fluid pressure, internal energy, fluid dynamic viscosity, fluid thermal conductivity, and the fluid velocity, respectively. Moreover, $C_{v}$ (J/k), Φ, $S_{M}$, $S_{E}$ and *R* (8.314 J/(mol·k)) correspondingly refer to the heat capacity, dissipation equation, momentum source, energy source, and the ideal gas constant, respectively. In this research, an initial investigation of the Reynolds number (Re) was conducted, revealing that the flow regime remains laminar within the designated SHGFR range (30–120 sccm). Consequently, a laminar viscous model was selected for further analysis.

#### 3.2.2. Discrete Phase Modeling

When the input gas flow reaches a steady state, DPM is then employed to trace particle trajectories upon their exit from the nozzle, utilizing the following equation to analyze the aerosol particle movement [31]:

(6) $$\overset{⃑}{F}=V_{d}\cdot \rho_{d}\frac{d{\overset{⃑}{v}}_{p}}{dt}$$

Moreover, due to the small size of aerosol particles in AJP, only the drag force, Saffman lift force, and gravity are examined within the model. Hence, the combined force ($\overset{⃑}{F}$) acting on droplets can be formulated as shown in the following [32]:

(7) $$\overset{⃑}{F}={\overset{⃑}{F}}_{D}+{\overset{⃑}{F}}_{Saff}+{\overset{⃑}{F}}_{g}$$

**Figure 3:**
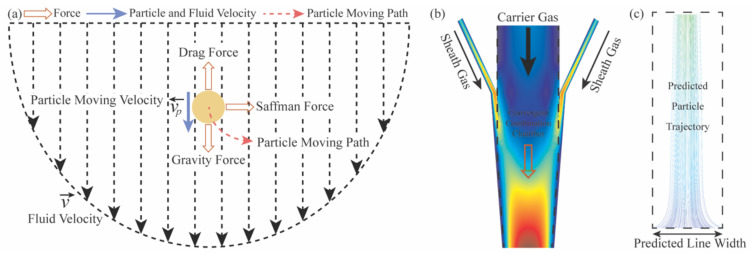
(**a**) The main forces acting on droplets as they pass through a shear gas flow, (**b**) DPM-based particle tracking, and (**c**) Numerical measurement of the printed line width in AJP.

Figure 3a represents the resultant force $\vec{F}$ applied to the droplets as they traverse a shea gas flow. Specifically, the drag force ${\overset{⃑}{F}}_{D}$, gravity force ${\overset{⃑}{F}}_{g}$ and Saffman lift force ${\overset{⃑}{F}}_{Saff}$ are expressed as follows [33]:

(8) $${\overset{⃑}{F}}_{D}=\frac{3\mu C_{D}Re}{4\rho_{D}d_{D}^{2}},\;\;\;Re=\frac{\rho d_{p}}{\mu }\left(\overset{⃑}{\nu }-{\overset{⃑}{\nu }}_{p}\right)$$

(9) $$F_{Saff}=1.61\mu d_{D}\left|\overset{⃑}{\nu }-{\overset{⃑}{\nu }}_{p}\right|\sqrt{\frac{d_{u}}{d_{y}}\frac{d_{D}^{2}}{\gamma_{c}}}$$

(10) $${\overset{⃑}{F}}_{g}=mg$$

where $\vec{v_{p}}$ (m/s), μ ($m^{2}/s$), $\gamma_{c}$ ($m^{2}/s$), $V_{d}$ (m^3^), $\vec{v}$ (m/s), and $\rho_{d}$ (kg/m^3^) are the particle moving velocity, fluid kinematic viscosity, kinematic viscosity, particle volume, fluid velocity and the particle density, respectively. $d_{u}/d_{y}$ is the velocity gradient (1/s) and $d_{p}$ (m) and $d_{D}$ are the channel and particle diameters, respectively. Moreover, the drag force coefficient $C_{D}$, expressed as $C_{D}=a_{1}+\frac{a_{2}}{Re}+\frac{a_{3}}{Re}$, relies on constants ($a_{1}$, $a_{2}$, and $a_{3}$) determined according to the Reynolds number [34].

When the fluid velocity exceeds that of the droplets, there is a potential for droplet breakup as the droplets exit the tip. Therefore, the TAB model is utilized to examine this phenomenon. In the context of the TAB model, there is an assumption regarding the equivalence of distortion and oscillation,31 which is mathematically described from Equations (11)–(14) following [35]:

(11) $$\overset{⃑}{F}-kx-d\frac{dx}{dt}=m\frac{d^{2}x}{dt^{2}}$$

(12) $$\frac{d}{m}=C_{d}\frac{\mu_{1}}{\rho r^{2}}$$

(13) $$\frac{\overset{⃑}{F}}{m}=C_{F}\frac{\rho {\overset{⃑}{v}}^{2}}{\rho_{p}r}$$

(14) $$\frac{k}{m}=C_{k}\frac{\sigma }{\rho r^{3}}$$

In these equations, $C_{d}$, $C_{F}$, and $C_{k}$ are constants obtained from the literature [31]; r denotes the droplet radius in meters; x is the displacement of the droplet from its spherical (undisturbed) position; and the corresponding critical value x>0.5r can be used to determine if the droplet will break up as the distortion grows.

Figure 3b demonstrates the tracking of particle paths by integrating a state of force equilibrium on them from the printhead using the DPM. Subsequently, upon exiting the nozzle, as depicted in Figure 3c, the width of the calculated particle trace can be utilized for a numerical measurement of the deposited line width in AJP. The experiments were then compared to the obtained numerical outcomes to validate the accuracy and reliability of the adopted model.

### 3.3. Model Assumptions and Boundary Conditions

In this study, the CFD simulation utilizes a timestep of 10^−5^ s, operating on the following foundational assumptions:

(1) There is no evaporation of droplets during the printing process.
(2) Droplet sizes are uniform, and collisions between droplets are absent during their transport and deposition phases.
(3) Material properties remain consistent throughout ink transport and deposition processes.
(4) Both the environmental and fluid temperatures remain constant.

Moreover, the numerical model in this research is defined further by the following specific boundary conditions [21,22]:

(1) The CGFR and SHGFR are set as the mass flow rate input and velocity input, respectively.
(2) The walls in the continuous phase are stationary with a no-slip condition. In the discrete phase modeling step here, the nozzle interior surface was set to reflect droplets, while the substrate was set to trap droplets.
(3) The initial velocity of the atomized ink droplets aligns with the carrier gas flow velocity.
(4) Gas exiting the nozzle towards the substrate in the continuous phase uses an atmospheric pressure outlet condition, while the discrete phase employs an escape condition at the pressure outlet.

## 4. Results and Discussion

The influence of different gas flows on the deposited line width are investigated in this section investigates the influence of CGFR and SHGFR on the width of printed lines. The numerical results are also verified with the printed line morphology and the corresponding line electrical performance.

### 4.1. Influence of the CGFR on the Printed Line Width

**Figure 4:**
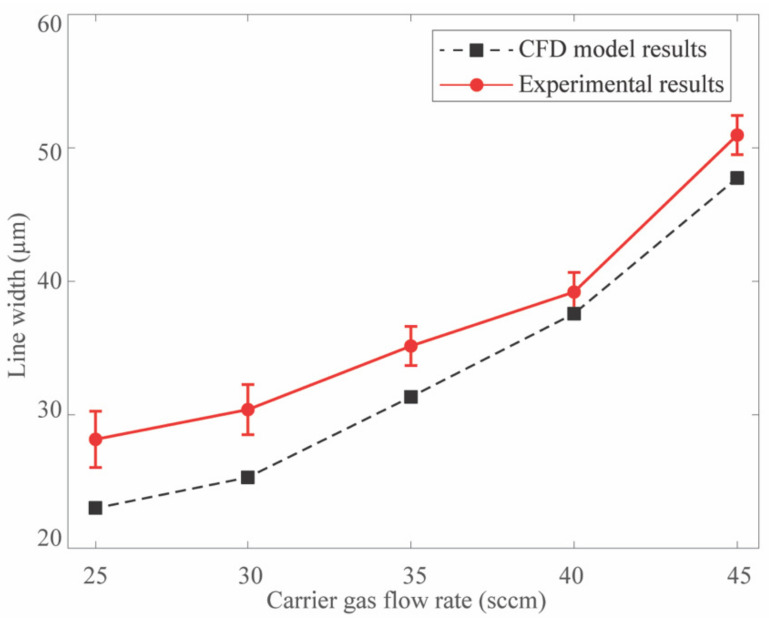
Comparison of simulation results with experimental findings regarding the deposited line width across varying CGFRs.

To analyze the influence of the CGFR on the velocity profile and the corresponding droplet movement in the printhead, single-factor experiments were designed, with the CGFR ranging from 25 to 45 sccm while maintaining the SHGFR at 60 sccm. In this research, the droplet size is fixed at 3 µm, adhering to recommended sizes in the range of 1–5 µm [25,36]. This study analyzed the variations in the flow field and pressure field on the axisymmetric plane of the printing nozzle under the influence of operating parameters. The results showed that increasing CGFR leads to wider line widths due to diminished collimation. Figure 4 compares the experimental outcomes (denoted by the red solid line) with the numerical predictions (black dotted line), demonstrating a consistent overall trend of line width expansion with previous research using a 2D CFD model with respect to higher CGFRs. However, discrepancies (less than 20%) exist between the experimental and simulation results, likely due to factors such as non-uniform droplet sizes, droplet collisions during transport and deposition, liquid evaporation influenced by the high gas flow rates, and complex dynamics such as temperature shifts and random variations during printing. Consequently, the interplay of these influencing parameters could jointly account for the disparities obtained between the simulations and the experimental findings.

**Figure 5:**
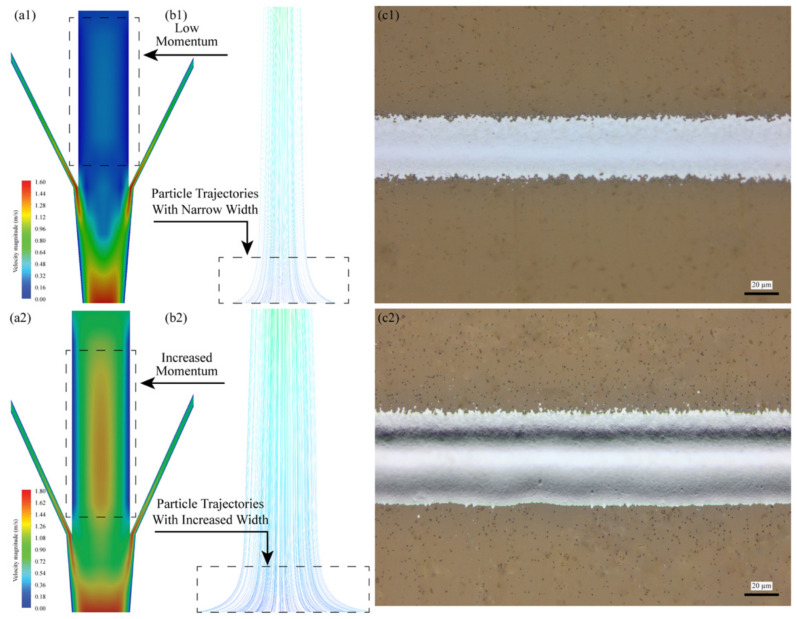
Numerical model analysis across different CGFR conditions (SHGFR = 60 sccm): (**a1**,**a2**) obtained momentum of atomized ink droplets and velocity profiles at corresponding CGFRs of 25 sccm, and 45 sccm, (**b1**,**b2**) obtained particle trajectories corresponding to (**a1**,**a2**), and (**c1**,**c2**) respective printed line samples obtained under the working conditions in (**a1**,**a2**).

Determining the influence of the CGFR on the aerosol stream width within a print channel, particularly after the stream exits the nozzle where there is no wall confinement, is crucial for understanding particle collimation variations. Figure 5 analyzes the influence of varying the CGFR on the velocity profiles in a CFD-simulated mist tube. Specifically, the comparison between Figure 5a1,a2 demonstrates that a higher CGFR increases the momentum of atomized ink droplets within the tube, further validating the significant interactions between SHGFR and CGFR within the printhead, which is in accordance with previous research using a 2D CFD model. Consequently, Figure 5b1,b2 shows that an increased CGFR results in a wider line width. This occurs because a higher CGFR reduces the relative velocity between the particles and the fluid, diminishing the Saffman force and, thus, broadening the line width. Additionally, increased CGFR boosts the evaporation rate of the droplets, further decreasing their diameter and Saffman force at the nozzle exit. This combined effect allows the aerosol flow to better resist collimation, leading to wider lines at elevated CGFR levels. Figure 5c1,c2 experimentally validate the effects of the CGFR on the deposited line width.

### 4.2. Influence of the SHGFR on the Printed Line Width

**Figure 6:**
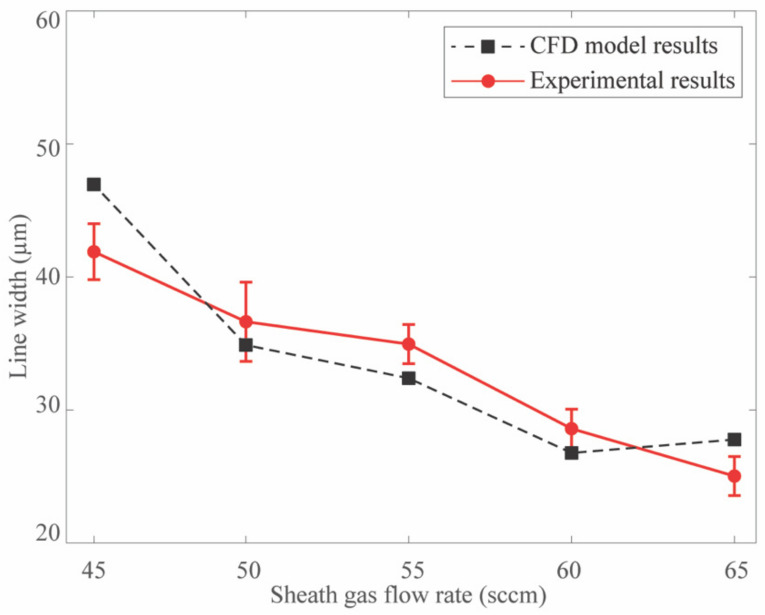
Comparison of simulation results with experimental findings regarding the deposited line width across varying SHGFRs.

Previous research highlights the substantial aerodynamic interactions induced by the sheath gas flow within the deposition head entering the combination chamber. However, a more in-depth investigation is needed to understand the impact of the SHGFR on the velocity profile and subsequent droplet collimation when the flow exits the nozzle [37]. Therefore, this section explores the influence of varying the SHGFR on velocity profile, droplet collimation, and corresponding line width. Specifically, the SHGFR is adjusted from 45 sccm to 65 sccm in 5 sccm increments. Figure 6 illustrates the impact of SHGFR variations on the printed line width, comparing the experimental data (red solid line) with the numerical prediction outcomes (black dotted line). The obtained results suggest that increasing the SHGFR narrows the line width due to improved particle focusing and collimation in the converging print channel, demonstrating a consistent overall trend with previous research using a 2D CFD model regarding increased SHGFR. On the other hand, the developed CFD model lacks consideration of ink-substrate interactions and droplet behavior, resulting in deviations (less than 20%) from the experimental data. Despite this, the simulation aligns with experimental trends and can compensate for insufficient experimental data.

**Figure 7:**
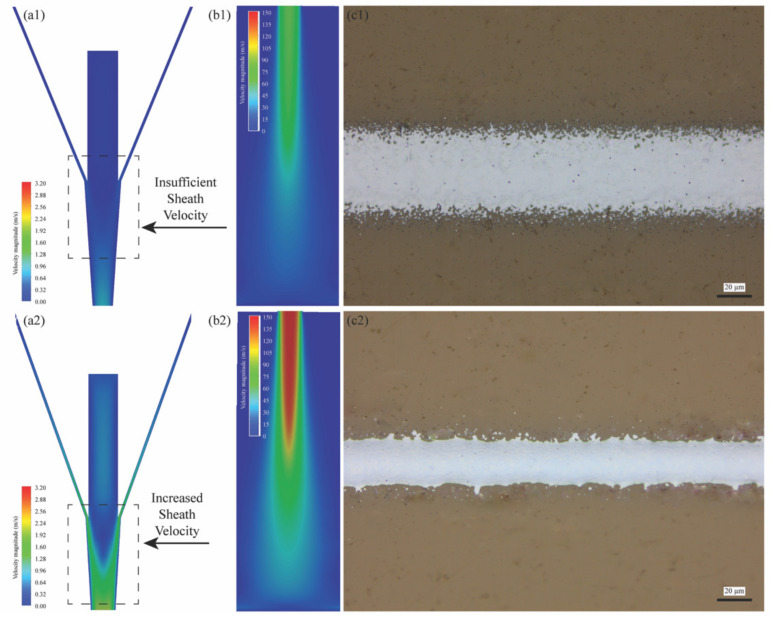
Numerical model analysis across different SHGFR conditions (CGFR = 35 sccm): (**a1**,**a2**) observed velocity profiles of ink droplets at corresponding SHGFRs of 45 sccm and 65 sccm, (**b1**,**b2**) obtained velocity contours of ink droplets upon exiting the nozzle corresponding to (**a1**,**a2**), and (**c1**,**c2**) respective printed line samples obtained under the working conditions in (**a1**,**a2**).

Figure 7 presents the results from the developed CFD model that examines the effects of varying SHGFRs on the fluid dynamics in the printhead. As illustrated in Figure 7a1,a2, increasing the SHGFR results in higher velocity profiles in the combination chamber, with the gas flow accelerating further through a converging channel, particularly at higher SHGFRs. Subsequently, as shown in Figure 7b1,b2, the fluid exhibits increased divergence after exiting the nozzle due to the reduced wall confinement, an effect more pronounced at higher SHGFRs. However, the particle trajectory becomes more collimated with an increase in the SHGFR, primarily due to the dominant Saffman lift force, which is stronger with higher fluid velocity gradients and relative droplet velocities. This effect leads to narrower printed lines, as confirmed by the experimental data in Figure 7c1,c2, where lower SHGFRs result in wider lines due to diminished Saffman force and increased particle divergence. On the other hand, it should be noted that the velocity field of the developed 3D model, upon exiting the nozzle, is significantly higher than that of a corresponding 2D model, indicating intensified Saffman lift force exerted on particles and greatly altered behavior subsequent to ejection.

### 4.3. Impacte of the CGFR and SHGFR on the Deposited Line Electrical Performance

**Figure 8:**
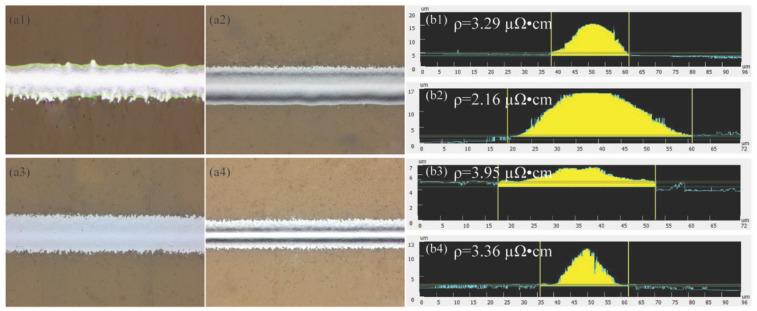
Influence of gas flow rates on the printed line electrical performance: (**a1**,**a2**) line samples printed at a low CGFR and a high CGFR, respectively (SHGFR = 50 sccm), (**a3**,**a4**) line samples printed at a low SHGFR and a high SHGFR, respectively (CGFR = 30 sccm), and (**b1**–**b4**) cross-sectional profiles of the printed lines corresponding to (**a1**–**a4**).

Figure 8a1,a2 show line samples printed at a low CGFR and a high CGFR, respectively. Specifically, the experimental results indicate that an increased CGFR within the print channel leads to greater momentum of the atomized ink droplets, resulting in diminished collimation and subsequently wider line widths. On the other hand, the effect of greater ink droplet accumulation further contributes to the formation of thicker lines at elevated CGFR levels, which is further validated in Figure 8b1,b2. To analyze the impact of the CGFR on the printed line electrical performance, the resistivity of printed line samples was measured using Ohm’s law

(15) $$R=\rho \frac{L}{S}$$

In this research, a four-point probe method was adopted to measure the printed line resistance (R) [11], the cross-sectional area (S) of each line sample (L = 1.0 cm) was measured three times and then averaged to calculate the printed line resistivity (ρ). The obtained resistivity for printed line samples at a low CGFR and high CGFR were 3.29 µΩ⋅cm and 2.16 µΩ⋅cm, respectively, demonstrating that increasing the CGFR leads to thicker lines and reduced resistivity owing to the greater amount of ink that accumulates and the increased line thickness.

On the other hand, Figure 8a3,a4 shows line samples printed at low and high SHGFRs, respectively. These results indicate that an increased SHGFR within the print channel has a greater focusing effect on the atomized ink droplets, resulting in narrower lines. Moreover, due to the mass continuity equation effect, a higher SHGFR also contributes to the formation of thicker lines, which is validated further in Figure 8b3,b4. To analyze the impact of the SHGFR on the electrical performance of printed lines, their resistivity was measured, with values decreasing from 3.95 µΩ⋅cm to 3.36 µΩ⋅cm as the SHGFR was increased, demonstrating that a higher SHGFR not only results in narrower but also thicker lines, thereby reducing the resistivity.

## 5. Conclusions

In this study, a 3D numerical model was developed to explore the intricate aerodynamic mechanisms within AJP. The proposed approach integrates CFD and DPM, offering a comprehensive understanding of the deposition mechanisms of the AJP process. Compared with a traditional 2D CFD model, the developed 3D CFD model can fully investigate the interactions between sheath gas and carrier gas within the printhead and further validate the overall trend of the printing process as provided by a 2D model with respect to different process parameters. The measured printed line morphology and corresponding line electrical performance exhibited close conformity with the numerical model, demonstrating that the proposed numerical model is important for making well-informed decisions, which can serve as guidance for improving process optimization efficiency and reducing waste during printing. However, the developed 3D CFD model did not take the ink properties, ink evaporation rate, and particle size into account. This may result in deviations between experiments and the modeling results. Therefore, in future research, it is essential to incorporate the dynamics of the ink properties, including the viscosity and surface tension, as well as the effects of ink evaporation and particle size variations. These factors are critical for accurately predicting the behavior of ink during the printing process.

## Figures and Tables

**Table 1:** Aerosol jet printing setup.

Operating Parameters			Working Conditions				
SHGFR	CGFR	Print Speed	Ink Temperature	Atomization Current	Working Distance	Tip Diameter	Plate Temperature
45–65 sccm	25–45 sccm	1 mm/s	20 °C	0.45 A	4 mm	150 μm	Ambient temperature

## Data Availability

The original contributions presented in the study are included in the article, further inquiries can be directed to the corresponding author.
